# The patterns and driving forces of dengue invasions in China

**DOI:** 10.1186/s40249-023-01093-0

**Published:** 2023-04-21

**Authors:** Zhe Zhao, Yujuan Yue, Xiaobo Liu, Chuanxi Li, Wei Ma, Qiyong Liu

**Affiliations:** 1 Department of Epidemiology, School of Public Health, Cheeloo College of Medicine, Shandong University, 44 Wenhua Road, Lixia District, Jinan, 250012 People’s Republic of China; 2 State Key Laboratory of Infectious Disease Prevention and Control, National Institute for Communicable Disease Control and Prevention, Chinese Center for Disease Control and Prevention, 155 Changbai Road, Changping District, Beijing, 102206 People’s Republic of China; 3 Department of Vector Control, School of Public Health, Cheeloo College of Medicine, Shandong University, 44 Wenhua Road, Lixia District, Jinan, 250012 People’s Republic of China; 4 Shandong University Climate Change and Health Center, Jinan, 250012 People’s Republic of China

**Keywords:** Dengue, Invasion, Imported case, Landscape, Driving forces, China

## Abstract

**Background:**

Global connectivity and environmental change pose continuous threats to dengue invasions from worldwide to China. However, the intrinsic relationship on introduction and outbreak risks of dengue driven by the landscape features are still unknown. This study aimed to map the patterns on source-sink relation of dengue cases and assess the driving forces for dengue invasions in China.

**Methods:**

We identified the local and imported cases (2006–2020) and assembled the datasets on environmental conditions. The vector auto-regression model was applied to detect the cross-relations of source-sink patterns. We selected the major environmental drivers via the Boruta algorithm to assess the driving forces in dengue outbreak dynamics by applying generalized additive models. We reconstructed the internal connections among imported cases, local cases, and external environmental drivers using the structural equation modeling.

**Results:**

From 2006 to 2020, 81,652 local dengue cases and 12,701 imported dengue cases in China were reported. The hotspots of dengue introductions and outbreaks were in southeast and southwest China, originating from South and Southeast Asia. Oversea-imported dengue cases, as the Granger-cause, were the initial driver of the dengue dynamic; the suitable local bio-socioecological environment is the fundamental factor for dengue epidemics. The Bio8 [odds ratio (*OR)* = 2.11, 95% confidence interval (*CI)*: 1.67–2.68], Bio9 (*OR* = 291.62, 95% *CI*: 125.63–676.89), Bio15 (*OR* = 4.15, 95% *CI*: 3.30–5.24), normalized difference vegetation index in March (*OR* = 1.27, 95% *CI*: 1.06–1.51) and July (*OR* = 1.04, 95% *CI*: 1.00–1.07), and the imported cases are the major drivers of dengue local transmissions (*OR* = 4.79, 95% *CI*: 4.34–5.28). The intermediary effect of an index on population and economic development to local cases via the path of imported cases was detected in the dengue dynamic system.

**Conclusions:**

Dengue outbreaks in China are triggered by introductions of imported cases and boosted by landscape features and connectivity. Our research will contribute to developing nature-based solutions for dengue surveillance, mitigation, and control from a socio-ecological perspective based on invasion ecology theories to control and prevent future dengue invasion and localization.

**Graphical Abstract:**

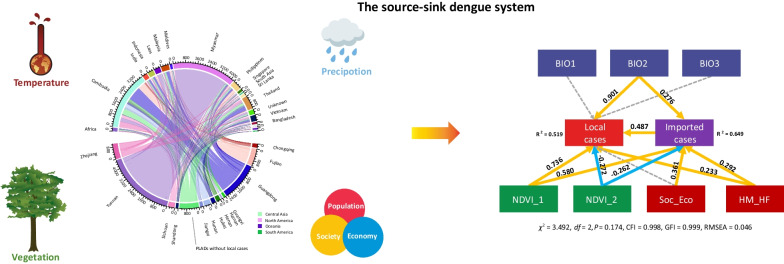

**Supplementary Information:**

The online version contains supplementary material available at 10.1186/s40249-023-01093-0.

## Background

Dengue is a globally spreading arboviral disease, whose endemic cases have expanded to 129 countries [1]. The most severely affected dengue hotspots are mainly clustered in Southeast Asia, the Western Pacific regions, and the Americas, of which Asia contributes 69.58% of the disease burden worldwide [2, 3]. In 2019, the largest dengue pandemic in history occurred, with a total number of 5.2 million cases [2]. The coronavirus disease 2019 (COVID-19) pandemic has partly disrupted dengue transmission, causing overall cases to decline due to policy interventions and human mobility restrictions during the Public Health Emergency of International Concern [4]. However, in 2022, more than 2.8 million dengue cases were confirmed in the Americas, indicating a return to normal levels compared to the levels before the COVID-19 pandemic (2,893,962 in 2016 and 3,181,171 in 2019) [5]. Dengue remains a significant global health concern that requires sustained efforts to prevent exacerbated devastating consequences coupled with the COVID-19 pandemic.

Primarily borne by *Aedes aegypti* and *Aedes albopictus*, dengue virus (DENV) transmission is sensitive to environmental conditions and constrained to abiotic resources due to the biological traits of the pathogen-vector-host nexus [6, 7]. However, human activities have triggered significant environmental changes, breaching four of the nine planetary boundaries, and potentially altering the ecology of infectious diseases [8–10]. Climate change, phenology change, alien species invasion (the geographic expansions of DENV, *Ae. aegypti* and *Ae. albopictus*), and their cascading nexuses could boost the global dengue spread, which challenges the vector and dengue control [7, 11]. Meanwhile, two parallel dengue transmission patterns could be observed due to the amplification effect of global connectivity when focused on the country levels under the local environmental condition changes. One is the indigenous cases limited to country boundaries induced dengue endemic spreading, and the other is the exogenous case invasions that caused the local transmissions and attempted to establish its own endemic cycle in a new suitable environment. Thus, understanding dengue's invasion, colonization, and spreading processes is crucial, especially in countries with widely distributed dengue vectors without dengue endemics.

In China, it is widely believed that previous dengue epidemics have been caused by local transmission induced by imported cases, which suggests that the DENV bio-geographical invasions and circulations could be established via international transit routes [7, 12]. The COVID-19 pandemic has provided a natural experiment for understanding the localization of dengue in China, further confirming that the imported cases are the initial driver leading to dengue transmission [4, 13]. Since COVID-19 has been treated as a Category B disease to manage in China, the invasion and transmission of DENV might be ‘reburned’ as the lifted restrictions on international travel and local-scale human mobility. However, previous studies have focused on the dynamics and drivers of dengue outbreaks after DENV introductions, which may neglect the roles that imported cases, local cases and the nexus to the environmental drivers play in the macrosystem. In order to make sufficient public health preparedness in source control, it is essential to detect the source-sink patterns for the nexus of introduction-outbreak and assess the driving forces of dengue transmission in the systemic view.

Here we drew the pattern of the source-sink relationship of dengue cases with both the imported and local epidemiological data from 2006 to 2020. We further confirmed the association between imported and local cases using the econometric methods based on chronological order relations. We also analyzed the correlations between dengue cases (including both imported and local information) and the environmental drivers and assessed the contributions of these drivers to the dengue endemic transmissions using a machine learning algorithm. We identified the major driving forces of the local dengue transmission and curved their exposure–response relationships. Finally, we reconstructed the internal connections among imported cases, local cases, and external environmental drivers using structural equation modeling (SEM) to detect the interlinkages in the dengue source-sink system. Our study will help to provide nature-based solutions for the surveillance, mitigation, and control of dengue from a socio-ecological perspective rooted in the invasion ecology theories.

## Methods

### Data on dengue cases

The dengue cases were sourced from the National Notifiable Infectious Disease Surveillance System. The clinically diagnosed and pathogen-confirmed dengue cases from 1 January 2006 to 31 December 2020 were collected, covering the geographic addresses (including source and current addresses), genders, and ages. The origin sources of dengue cases were classified as local, overseas imported, and domestic imported dengue cases. Local dengue cases were defined as those who resided in certain cities without leaving during the premorbid 14 days, with a bite history. Patients traveling from the dengue-endemic countries or regions within 14 days were categorized as overseas imported dengue cases, with a bite history. Cases that migrated from dengue-endemic cities and immigrated to the cities without dengue outbreaks premorbid 14 days were classified as domestic imported dengue cases, with a bite history[14]. We recounted the domestic imported cases into their original migrated geographic units and merged them into the local case group. The total dengue amounts of imported and local groups were summarized at town levels by the information provided by the geocodes of the cases.

### Data on environmental drivers

The gridded data for environmental drivers covered the information in five dimensions: climate, phenology, vegetation coverage, social development, and human activities (Additional file 1). The climate data contained 19 bioclimate variables (Bio1–Bio19), which were generated from the monthly temperature and rainfall data to show the biological meaning for a particular species, and were from the Chelsa Climate Dataset (https://chelsa-climate.org/) [15].

The phenology-related data was obtained from the Chelsa Dataset, which contained five covariates: growing degree days heat sum above 10 ℃ (GDD10), climate moisture index (CMI), accumulated precipitation amount on growing season days TREELIM (GSP), mean temperature of the growing season TREELIM (GST), and the number of growing degree days above 10 ℃ (NGD10), which are associated the biological traits of DENV-mosquitoes-host nexus.

The vegetation coverage dimension included two categories: net primary productivity (NPP) and normalized difference vegetation index (NDVI), both of which are relevant to mosquito biology. The NPP obtained from the Chelsa dataset shows the energy flows other organisms can use in the ecosystem. The NDVI from the dataset entitled ‘A 5 km resolution dataset of monthly NDVI product of China (1982–2020)’ was provided by the National Earth System Science Data Center (http://www.geodata.cn/). We aggregated the annual and monthly averages of NDVI.

The social development dimension was represented by gross domestic product (GDP) and population distributions. The population count data ‘Gridded Population of the World: Population Count, v4.11’ was sourced from the Socioeconomic Data and Applications Center (https://sedac.ciesin.columbia.edu/data/collection/gpw-v4/sets/browse) [16]. The GDP in 2015 was extracted from the dataset published from the Scientific Data to show the regional economic development [17]. To estimate the impacts of human activities on the terrestrial land system, the human modification index (HMI) was generated from 13 anthropogenic stressors (https://sedac.ciesin.columbia.edu/data/set/lulc-human-modification-terrestrial-systems) [18, 19]. The mean human footprint index (HFI) from 2000 to 2018 was calculated via the dataset published in Scientific Data to indicate the pressures imposed on natural landscapes and ecological processes driven by human activities [20].

We calculated the geometry centroids of the town-level-based administrative areas to obtain the geographical coordinates. The values of the environmental drivers were then extracted by these geometry centroid coordinates. To avoid the spatial sampling bias caused by a single sample point, a 10-km-radius buffer analysis was performed via the coordinates of the towns, which enabled us to calculate the average grid values of environmental drivers in these circle buffers.

### Descriptive analysis on the source-sink relationship of dengue in China

The total dengue occurrences at the city levels from 2006 to 2020 were aggregated separately in the local and imported case groups. In each group, cities without reporting any dengue cases were categorized as one specific cluster; for the cities reporting any dengue cases, the Jenks natural breaks classification method [21], aiming to minimize the variance within the group and maximize the variance between groups, was applied to categorize the cities into three clusters. Thus, four clusters were shown in each group. We combined the two groups with four clusters and formed the 4 × 4 matrix to draw the source-sink relationship of dengue spatial distributions on the Choropleth Map. The R package ‘classInt’ was applied to perform the natural breaks classification method. The source-sink flows between the dengue exported countries and their imported provincial-level administrative divisions (PLADs) in China were counted. The proportions of seasonal imported and local cases were summarized. The epidemiological characteristics of local dengue cases, including gender and age distribution, were sorted based on PLADs. The R packages ‘circlize’ and ‘ggalluvial’ were loaded to perform the high-dimensional visualized methods.

### Modeling the relationship in imported-local dengue cases

The monthly time-series data for imported and local dengue cases from 2006 to 2020 were aggregated at the country-level and scaled with logarithm. The Spearman correlation test was applied to detect the relation between local and imported series (source-sink relation). The robust statistical properties of the time series are the prior condition to avoid spurious regression in modeling procedures. The augmented Dickey-Fuller test, a commonly used unit root test, was applied to check the stationarity of the time series. The Granger test was performed to statistically confirm the causality between local and imported dengue cases in the source-sink system. The vector autoregressive model (VAR) was built to identify the cross-correlation of the dengue source-sink system. The lag order for endogenous variables is crucial for the accuracy and reliability of the model, which was balanced according to the Akaike information criteria (AIC) to avoid overfitting and underfitting. The 2-order lag was selected based on AIC during the following tests and modeling procedures. An empirical fluctuation process according to the generalized fluctuation test framework was computed to check the stability of VAR. The Ljung-Box test was applied to verify the randomness of the residuals of VAR to ensure the model has extracted the time-dependent information in source-sink patterns in the time series. To check whether the crucial information of the dengue source-sink system is covered and improve the accuracy of the estimates, we detect the heteroscedasticity effect of the residuals in VAR by the auto-regressive conditional heteroskedasticity with Lagrange multiplier test. The impulse response coefficients and 95% confidence intervals (*CI*) were computed with 1000-run bootstrap to show the shocks that other system components imposed on one specific component in the dengue source-sink system. We computed the forecast error variance decomposition of VAR to show the contribution of structural shocks to changes in endogenous variables. The R package ‘urca’ and ‘vars’ were applied during the time-series analysis.

### Modeling the importance of environmental drivers to local cases

We aggregated the mean values of buffered environmental variables at the city level and counted their local and imported cases. The Spearman correlation coefficients were calculated to show the co-relation of the environmental drivers. The Mantel test, which combined with the Spearman and permutation test, were applied to determine the correlations between dengue source-sink system and environmental drivers [22]. The variable selections were applied by a machine learning method to examine the contributions of the drivers. Random forest (RF) is a classical algorithm for classification and regression, which could provide high performance in predictive accuracy during variable selection procedures [23–25]. The ‘logit’ linkage function was structured to model the city-level presence/absence status of local dengue cases in the RF. The data was split into two parts to avoid overfitting: the training set (70%) and the test set (30%). In each set, the ratio of presence/absence is accorded to these in the initial dataset. The number of trees to grow (ntree) and the number of variables randomly sampled as candidates at each split (mtry) is the two main parameters for the RF performances [25]. The grided search was applied to tune the optimal parameter combination evaluated by the accuracy. The ranges of mtry and ntree are 2–20 (step = 1) and 400–4000 (step = 100) with 361 combinations. The tenfold cross-validation was performed to boost the stability of modeling. The final optimal tuned parameters (ntree = 1000, mtry = 5) were applied in the variable selection procedure. The R packages ‘tidymodels’ and ‘randomForest’ were loaded to model and tune the hyperparameters of RF.

The vast amount of variables, which may be irrelevant to the classification in feature selection and unknown in advance, could lead to the curse of dimensionality, which could intervene in the accuracy [26]. The Boruta algorithm was developed to improve the feature selection during the machine learning process by comparing the relevance of the real features to the random probes [26]. It shuffled the features into shadow features, trained the RF with these features, calculated their feature scores, and further tested by *Z*-score test. The algorithm repeats this process iteratively, until all features have been either confirmed or rejected. The maximal number of importance source runs was set to 3000. These procedures were powered by R packages ‘Boruta’.

### Modeling the exposure–response relationships to local cases

We construct the ‘linear-basis function’ combined with general additive models (GAM) to evaluate the exposure–response relationships to the local dengue epidemics. A ‘quasi-Poisson’ distribution was applied to deal with overdispersion. After confirming the contributions of selected variables to dengue local transmission using the Boruta algorithm, Spearman correlations were used to filter the variables and control for multicollinearity. Variables selected by the Boruta algorithm whose Spearman correlation coefficients are no more than 0.7 could be included for GAM fitting. The dengue epidemic risk association with major environmental driving forces was described using odds ratios (*OR*) and corresponding 95% *CI* by comparing the risk at the average levels of the cities without local dengue occurrences. R packages ‘splines’, ‘mgcv’ and ‘dlnm’ were used.

### Modeling the structure of the dengue driving forces

To comprehensively understand the mechanisms of the dengue source-sink system and identify the association rules among landscape compositions, a SEM was constructed to analyze the interlinkage among imported cases, local cases, and environmental drivers. Principal components analysis (PCA) was taken to control the multicollinearity. The optimal model was selected with minimum AICc, using the function ‘aictab.lavaan’ created by Grace in the R software [27]. The ‘psych’ and ‘lavaan’ packages of R software were applied during the procedure.

All analysis was performed within R 4.2.0 (R Foundation for Statistical Computing, Vienna, Austria).

## Results

### The source-sink relationship of dengue in China

**Fig. 1:**
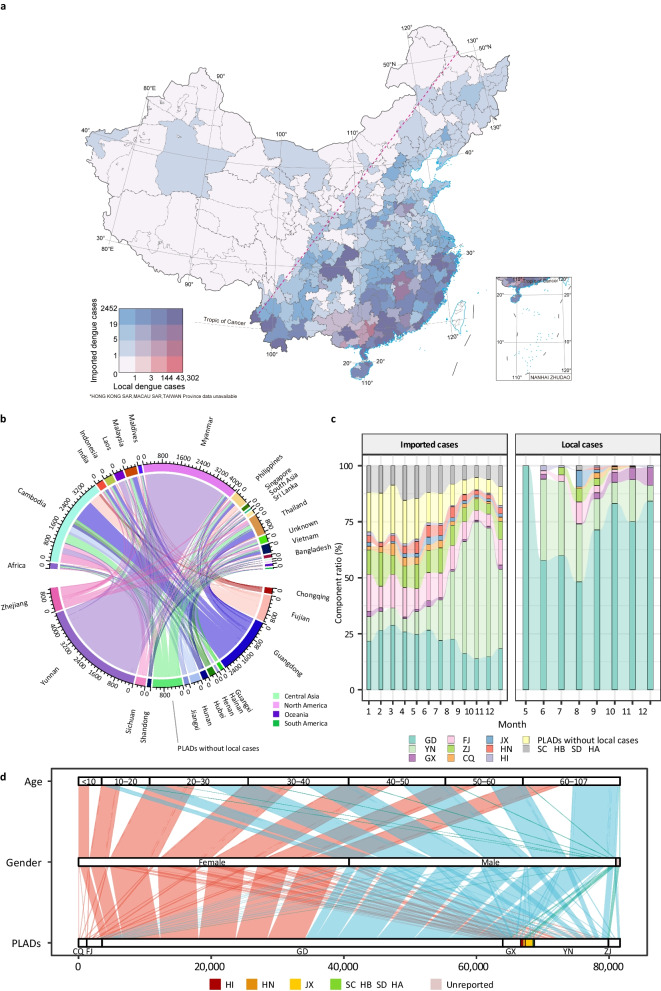
The source-sink relationship of dengue in China. **a** The distributions of oversea dengue introductions and local outbreaks. The cities with local dengue cases indicate that dengue outbreaks have occurred. There are three natural breakpoints in captions: 1, 23, and 144, dividing the square four into parts to show the gradient levels of the risks with warm colors. The cities with imported dengue cases indicate dengue introductions from overseas. There are three natural breakpoints in captions: 1, 5, and 19, dividing the square four into parts to show the gradient levels of the risks with cold colors. The 4 × 4 grids show the city levels of source-sink risks. The red dashed line is Hu Line, a geographical demarcation line for population distribution and economic development in China. **b** The interlinkages between source regions/countries and the sink provincial-level administrative divisions (PLADs) in China of dengue imported cases. The dengue source-sink flows between the exported countries and the imported PLADs in China were counted. **c** The monthly dengue cases (imported and local) component ratios at PLADs. The figure on the left was to show the seasonality changes in dengue oversea introductions. The figure on the right was to indicate the seasonal locally acquired dengue cases. GD is short for Guangdong, FJ for Fujian, JX for Jiangxi, YN for Yunnan, ZJ for Zhejiang, HN for Hunan, GX for Guangxi, CQ for Chongqing, HI for Hainan, SC for Sichuan, HB for Hubei, SD for Shandong, and HA for Henan. **d** The epidemiological characteristics (genders and age groups) of local dengue cases at PLADs. The intrinsic flows and counts among local dengue cases, genders, and age groups at PLADs were shown

From 2006 to 2020, 81,652 local dengue cases and 12,701 imported dengue cases were reported in China. The pattern of dengue distribution was mapped to show the three types of hazard points of integrated information on the imported-local nexus of reported cases (Fig. 1a). Cities that only reported imported cases were classified to display the cross-border transmission risks for dengue invasion. These regions, such as Jiangsu Province, could be transformed into the bridgehead for dengue spread when the ecological, hygienic, and quarantinable conditions change. Cities with more local cases and fewer oversea imported cases, such as some regions in Guangxi Zhuang Autonomous Region, indicated the spillover effects of the dengue transmission triggered by space-neighboring epidemic regions. Regions with high numbers of imported and local dengue cases were considered the cross-border imported cases resulting in local transmissions and colonization, such as the Pearl River Delta region in Guangdong province and southwest Yunnan, the bridgeheads for dengue invasion in China.

The majority of imported dengue cases were from Southeast Asia (Fig. 1b). Myanmar, Cambodia, and Thailand were major foreign sources of dengue introductions. Yunnan, Guangdong, and Fujian Provinces were the important sink regions. The seasonality of the source-sink relationships at provincial levels was shown to clarify the regional time-scale vulnerability (Fig. 1c). Dengue transmission patterns showed regression with seasonality and phenology: earliest dengue outbreaks in Guangdong happened in May, north reached Shandong in August, and returned to Southeast China. Imported cases were reported in all months, but local transmission was restricted from May to December. No locally acquired cases were reported during the winter. As the transmission season was not enduring throughout the calendar year, the re-introduce of DENV was considered the initial factor for dengue dynamics in China. Two different patterns were detected in Guangdong and Yunnan. For imported cases, these two central sink PLADs were shown seasonality in peaks: ruggedness in Yunnan (boosted in the second half of the year) and smoothness in Guangdong (relatively higher in the first half of the year). The earliest local dengue outbreak occurred in May, happening in Guangdong. The epidemic peak in Yunnan was in summer, while it concentrates in autumn and early winter. The epidemiological characteristic in PLADs that experienced dengue outbreak were shown in the alluvial diagram (Fig. 1d).

### The interlinkage between oversea-imported and local dengue cases

To further detect the mutually consistent association between the source and sink in the system view, we analyzed the time-series data using the econometric approach to show how the synergic effect plays in dengue dynamics.

**Fig. 2:**
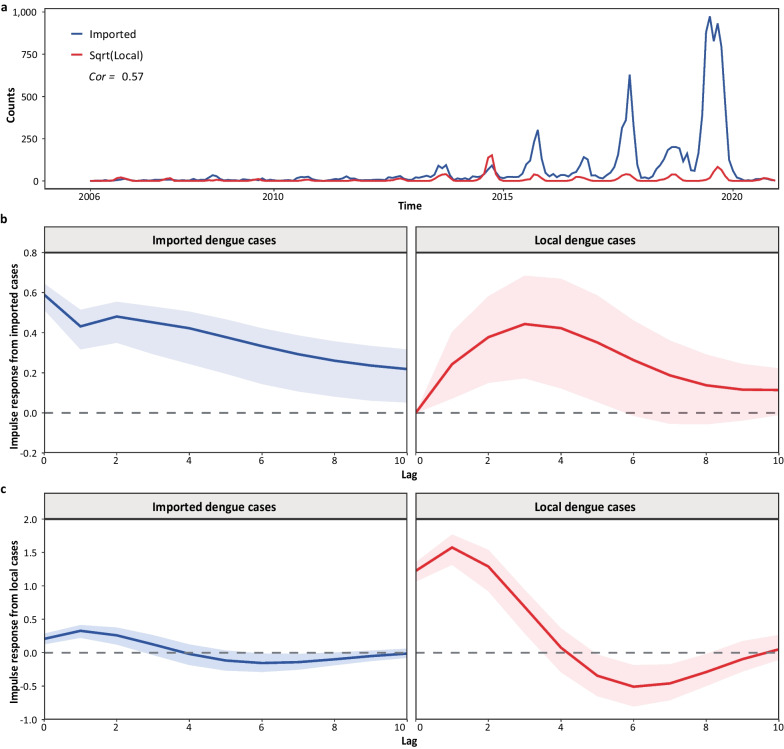
The interlinkage between oversea-imported and local dengue cases. **a** The monthly time-series on imported and local dengue cases in China. The Spearman correlation was 0.57, *P*-value < 0.01. **b** Impulse response from imported cases to imported and local dengue cases. The shocks imposed from imported cases to the dengue source-sink system were shown. **c** Impulse response from local cases. The shocks imposed from local cases to the dengue source-sink system were shown. The shadow areas show the 95% confidence intervals

The contemporaneous correlation between imported and local dengue cases in China was detected (Fig. 2a, Spearman *r* = 0.57, *P* < 0.01). Based on the Granger causality test, we confirmed that oversea-imported dengue cases were the Granger-cause of local dengue transmissions. For the impulse from the imported cases, the maximum effect on imported cases was reached in the current period and decayed over time; the impulse for the local cases showed an inverted ‘U’ shape curve, peaking at a three-month lag (Fig. 2b). Sine-like waves were shown when considering the impulse response from local cases, indicating the potential seasonality (Fig. 2c). Compared to these shocks posed to the imported cases, the impact on local dengue cases was much more prominent. The variance decomposition showed the mutual shocks that contributed to the system (Additional file 1). Local cases contributed the most to the local dengue dynamics, with a contribution rate was above 89%; the impact of imported cases on the local dengue epidemic peaked at a two-month lag with a 22% contribution. This indicated that oversea-imported cases are the initial driver of the dengue dynamic, while the suitable local bio-socioecological environment is the fundamental factor for dengue epidemics.

Combinedly, the pattern shows that the oversea-imported cases function as the ‘sparks’. The shifts in periodical landscape and ecological conditions provided the suitable environment for initiating the ‘conflagrations’ of dengue dynamics.

### The associations and selections of the environmental variables

To explore the interlinkages between environmental variables and dengue cases (both local and imported), we adopted a landscape-ecological perspective and employed ecological and evolutionary approaches to examine the driving forces behind these associations.

**Fig. 3:**
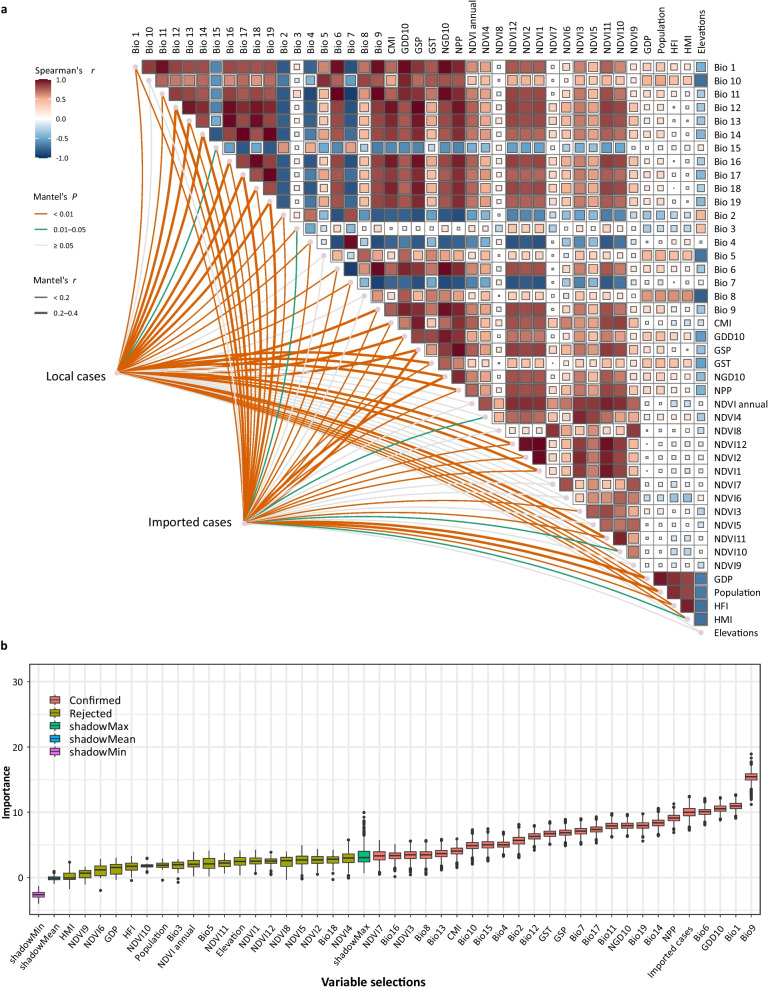
The initial associations of drivers in the dengue source-sink system and selections for the major driving forcings to the local dengue transmission. **a** The Spearman correlations on environmental variables and Mantel correlations of the environment-dengue nexus. The Spearman correlations were shown in the top right corner. The Mantel correlations were shown in the bottom left corner.** b** The contributions and selections of environmental drivers with Boruta algorithm. The selected variables were labeled as ‘Confirmed’ in the legend. ‘shadowMax’ was to show the cut-off showdown features between the ‘Rejected’ and ‘Confirmed’ variables. The following are the full names of the environmental drivers: NDVI: Normalized difference vegetation index; GDD10: Growing degree days heat sum above 10 °C; CMI: Climate moisture index; GSP: Accumulated precipitation amount on growing season days TREELIM; GST: Mean temperature of the growing season TREELIM; NGD10: Number of growing degree days above 10 °C; NPP: Net primary productivity; HMI: Human modification index; HFI: Human footprint index; Bio1: Mean annual air temperature; Bio2: Mean diurnal air temperature range; Bio3: Isothermality; Bio4: Temperature seasonality; Bio5: Mean daily maximum air temperature of the warmest month; Bio6: Mean daily minimum air temperature of the coldest month; Bio7: Annual range of air temperature; Bio8: Mean daily mean air temperatures of the wettest quarter; Bio9: Mean daily mean air temperatures of the driest quarter; Bio10: Mean daily mean air temperatures of the warmest quarter; Bio11: Mean daily mean air temperatures of the coldest quarter; Bio12: Annual precipitation amount; Bio13: Precipitation amount of the wettest month; Bio14: Precipitation amount of the driest month; Bio15: Precipitation seasonality; Bio16: Mean monthly precipitation amount of the wettest quarter; Bio17: Mean monthly precipitation amount of the driest quarter; Bio18: Mean monthly precipitation amount of the warmest quarter; Bio19: Mean monthly precipitation amount of the coldest quarter

The Spearman correlations on environmental variables and Mantel correlations of the environment-dengue nexus were mapped (Fig. 3a). Strong correlations were found among some environmental variables. Our analysis revealed strong correlations among some environmental variables, including climate, vegetation, phenology, and human activities, which may result in an overly simplified understanding of the features that contribute to dengue transmission in China if these factors are not properly accounted for in statistical models. The imported and local cases were associated with most of the environmental drivers, which demonstrates the socioecological traits of the dengue source-sink system.

The boxplots of the environmental drivers were used to display the distributions of the contributions of the variables during all selection procedures using the RF-based Boruta algorithm (Fig. 3b). The confirmed variables showed solid biological traits. For example, GDD10 (mean importance: 10.55%) is about accumulated temperature above 10 ℃, a crucial factor in mosquito biology; the imported cases (mean importance: 10.00%), confirmed in previous sections, were also detected during the feature discrimination. The area under curve (AUC) of RF was 0.965 for training-set and 0.943 for test-set.

### The major driving forces of dengue outbreaks

To explain the major driving forces behind local dengue transmission under an eco-epidemiological perspective, we fitted a regression model that included several variables: mean daily mean air temperatures of the wettest quarter (Bio8), mean daily mean air temperature of the driest quarter (Bio9), precipitation seasonality (Bio15), NDIV3, NDVI7, and oversea-imported dengue cases.

**Fig. 4:**
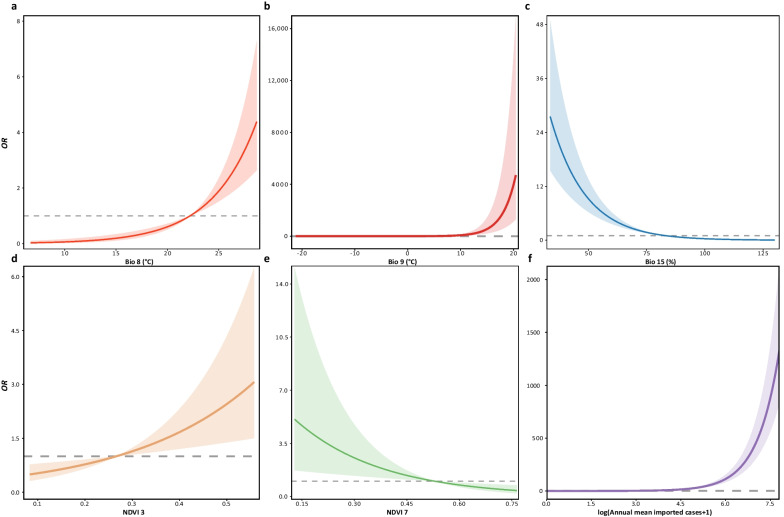
The major driving forces of dengue outbreaks and the exposure–response relationships. **a** The association between the mean temperature of the wettest quarter (Bio8) and local transmission of dengue; **b** The association between the mean temperature of the driest quarter (Bio9) and local transmission of dengue; **c** The association between the precipitation seasonality (Bio15) and local transmission of dengue; **d** The association between NDVI in March (NDVI3) and local transmission of dengue; **e** The association between NDVI in July (NDVI7) and local transmission of dengue; **f** The association between the annual mean imported cases at log scale and local transmission of dengue. The reference values on the exposure–response relationships were the average levels of the cities without dengue outbreaks (*OR* = 1), representing the relative risk-free points. The shadow areas show 95% confidence intervals

The positive association was represented between Bio8 and local transmission of dengue (Fig. 4a). Compared with the average level in cities without dengue occurrences (22.21 ℃, *OR* = 1), the average value of Bio8 in the cities with dengue occurrences (25.25 ℃) was posed a higher risk (*OR* = 2.11, 95% *CI*: 1.67–2.68). Similarly, when Bio9 reached the mean value (13.32 ℃) of the case group (Fig. 4b), the dengue risks were significantly increased (*OR* = 291.62, 95% *CI*: 125.63–676.89) compared to the reference level (− 1.04 ℃, *OR* = 1). This suggests that higher temperatures during both wetter and drier seasons were linked to dengue transmission. Additionally, we observed a positive association between dengue transmission and lower seasonal changes in precipitation, represented by Bio15 (Fig. 4c). When Bio15 was at 63.04%, the *OR* sharply increased to 4.15 (95% *CI*: 3.30–5.24), compared to the baseline of 83.38%.

A positive association between local dengue transmission and NDVI3 was found (Fig. 4d), with the *OR* reaching 1.27 (95% *CI*: 1.06–1.51), when NDVI3 reached the average level of the case group compared with the control group (NDVI3 = 0.27). In contrast, a negative association was detected between dengue risk and NDIV7 (Fig. 4e). The average level of NDVI7 in cities without dengue occurrences (0.53) was higher than those with local transmission. When NVDI7 reached the average level of dengue local transmission cities (0.52), the effect size was 1.04 (95% *CI*: 1.00–1.07), compared with the baseline (0.53). Based on the yearly averaged total amounts of oversea-imported records, dengue risk sharply increased with the invasion cases (Fig. 4f). The yearly averaged log-scaled accumulative imported cases in the control group were 0.38 (*OR* = 1), while the case group had a much higher risk with a value of 1.38 (*OR* = 4.79, 95% *CI*: 4.34–5.28).

### Identifying the structure of driving forces in dengue dynamics

To identify the association rules on the mechanism of dengue transmission dynamics in China, we composited SEM to deconstruct the nexuses of the landscape drivers at the ecological system level. The variable BIO2 was restricted by the variables closely associated with climate suitability for human niche and mosquito colonization (Additional file 1). NDVI_1 provided the information on NPP and the non-summer NVDI, while NVDI_2 presented the NDVI represented NDVI during the summer season and was applied to show the land use and land cover conditions. The variables labeled ‘Soc_Eco’ and ‘HM_HF’ contained loadings of GDP-population and HFI-HMI, respectively. The final model was validated by a robust-statistic test value of 3.492 with 2 degrees of freedom (df) and a *P*-value of 0.174.

**Fig. 5:**
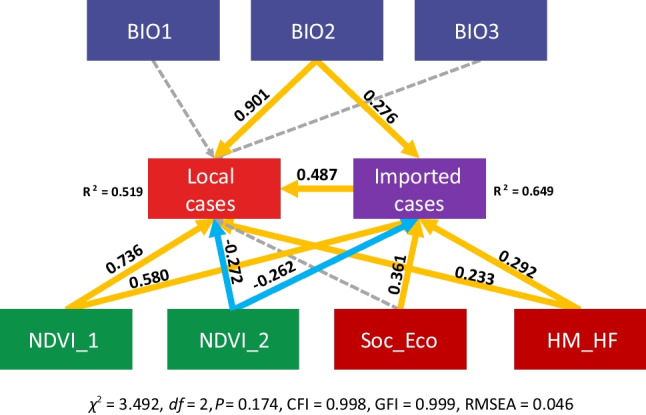
The structure of driving forces in dengue dynamics. NDVI_1 showed the information on NPP and the non-summer NVDI, and NVDI_2 presented the NDVI in the summer seasons, which were applied to show the land-use and land cover conditions. The variables labeled ‘Soc_Eco’ and ‘HM_HF’ contained loadings of GDP-population and Human Footprint-Human Modification Index. More information is provided in Additional file 1: Table S15–20. The final model was certified by a robust-statistic test value of 3.492 with 2 degrees of freedom (*df*) and a *P*-value of 0.174. CFI: comparative fit index (≥ 0.90), GFI: Goodness of fit index (≥ 0.90), and RMSEA: root mean square error of approximation (< 0.05)

NDVI_1 was a strong predictor of both local cases [standardized path coefficient (SPC): 0.736] and imported cases (0.580). NDVI_2 was a weak predictor of local cases (SPC: − 0.272) and imported cases (− 0.262). HM_HF was a weak predictor of both local cases (SPC: 0.233) and imported cases (SPC: 0.292). BIO2 was a strong predictor of local cases (SPC: 0.901) and a weak predictor for imported cases (SPC:0.276). Imported cases were associated with local cases (SPC: 0.487) and the intermediary variable for other environmental drivers. BIO1 and BIO3 were found to be independent of the source-sink system, while there was an intermediary effect for Eco_Soc on local cases via the path of imported cases (SPC: 0.361 × 0.487 = 0.176) (Fig. 5).

## Discussion

Identifying regions at risk for the introduction and spread of DENV and understanding the landscape drive forces is essential for tailoring strategies, preparedness, and response tactics while allocating scarce health and human resources. In this study, we mapped the source-sink combined patterns of the dengue imported-local cases and identified the hotspots of dengue epidemic clusters at the city scale caused by re-introduction. We confirmed that the dengue oversea-imported cases were the Granger-cause contributing to the dengue epidemics and found mutual-cross-series relations and long-run equilibrium between introductions and outbreaks of dengue in China. We found the major driving forces for the dengue landscape patterns under DENV invasion-localization processes and exposure-response relationships. Our findings showed the functions of drivers that contributed to the dengue outbreaks and detected the mechanistic structure of the dengue risk patterns in China from the landscape-based theory. Additionally, we detected that the intermediary effect on dengue dynamic system, where population and economic development contribute to local outbreaks via the path of imported cases in the dengue dynamic system. These findings could help with public health preparedness by providing natural-based solutions to dengue invasion and localization.

Our study detected the bridgeheads of dengue invasions and the founder effect at the city level. Since dengue outbreaks in China are induced by international travelers carrying DENV, it is essential to identify hotspots in the primary imported locations for optimized dengue source control [28]. Cities with oversea-imported cases are considered the primary introduction sinks and could establish footholds for further incursions and spreads [29]. Cities with more local cases and fewer oversea imported cases, such as some regions in Guangxi Zhuang Autonomous Region, could be driven unreported domestic imported cases and overseas-imported cases spillover from other regions. Guangxi is on the west of Guangdong Province, and shares borders with Cambodia and Laos, which are hotspots for dengue transmission and serve as the important source triggering dengue invasions. As a result, Guangxi is under double pressure from the spillover effects of dengue from these regions. From an alien species perspective, if suitable landscape conditions exist but there are no local dengue cases, a high frequency of intrusions and re-introductions may be necessary to accelerate the process of successful establishment. If local dengue transmission has occurred, indicating the establishment, outbreak, and spread of dengue in the region, it could serve as a foothold for the disease to spread to neighboring regions [29]. This could potentially result in a new invasion of dengue in those areas, making it function as a bridgehead that could trigger a cascading secondary introduction/spread process. Therefore, measures aimed at reducing the frequency of introduction, blocking invasion pathways, and eradicating spread should be implemented for the prevention of dengue outbreaks caused by imported cases.

The source-sink relationships suggest that public health preparedness should focus on the international connections among these regions to resile the expansion of dengue caused by increasing global connectivity. In the past twenty years, geographical distance has become less of a barrier to DENV dispersal due to the continuously increasing worldwide interconnections. International air travel is an important conduit to enhance global connectivity, thereby increasing dengue expansion risks to new regions [30]. The overall number of airline passengers has increased from slightly under two billion in 2000 to over four billion in 2019 [7]. This exponential expansion in international commercial airline networks poses continual threats to the spread of dengue in China [12, 28]. The immensely progressing human transport networks for economic and societal activities, where DENV is more likely to be introduced to and spread from highly connected hubs, can explain the high frequency of re-introductions, such as the recurrent epidemics in Guangdong [29, 31, 32]. In 2020, there were only two local cases of dengue reported in Guangzhou since there were fewer imported cases due to the lockdown [33]. Due to the COVID-19 being classified as a Class B infectious disease, dengue emergence might accelerate in the Pearl River Delta and spill over to the surrounding areas once the second generation of indigenous dengue cases is extensively reported. This indicates the wave of dengue introduction, colonization, and spread of dengue invasion [4, 33–35]. Our results illustrate that the dengue endemic in Southeast Asia was an important source region for dengue invasion, of which three countries that border China are Vietnam, Laos, and Myanmar. Ground transportation might be an option for international land travel in these regions, which could increase the flows across borders. Yunnan, another bridgehead for dengue introductions, faces intense pressure to prevent dengue invasion and localization as the only province that shares the borders with these three countries. Compared to the epidemics in the bordering countries over the preceding three years, dengue has been sharply down and sporadic in Yunnan due to the international travel restrictions [13]. As socioeconomic activities recover from the pressures of the COVID-19 pandemic, the challenges of dengue prevention and control might boost with the resumption of cross-border mobility, particularly for Yunnan, which serves as a major commercial hub for the Association of Southeast Asian Nations along the Belt and Road [4, 13

Our research demonstrated the link between imported and local dengue cases, based on time scale and seasonality. The time-lag effect was observed by the transmission of the impulse from imported cases to autochthonous cases which might be associated with dengue ecology. Dengue transmission is time-dependent process, as DENV needs to replicate in mosquitoes to acquire sufficient infectivity, and it also takes time to spread within the mosquito population and then to humans [6, 36, 37]. The self-impulse of local cases reflects the suitability of the local landscape conditions, which are probably impacted by climate factors affecting mosquito biology [38]. Eradicating a dengue outbreak once it has occurred can be time-consuming. The dynamic research in Zhanjiang, a city in Guangdong Province, showed a similar pattern to our results of the impulse-response relationship [39]. The first imported dengue case was reported in April and the first local case occurred in August, a four-month lag after the invasion. The outbreak ended on 28 October, three months after successful colonization. The time window for dengue control would be missed or delayed if the source of DENV infection was not promptly found, reported, and isolated. Therefore, health surveillance and transit quarantine at border entry must be implemented to screen for dengue or suspected patients to prevent introductions of imported cases. Our results suggested that dengue transmission showed a regression phenomenon in seasonality with the earliest dengue outbreaks in Guangdong occurring in May, expanding to Zhejiang in July, reaching Hubei, Henan, and Shandong in August, returning to Sichuan, Chongqing, Jiangxi, and Hunan, and then regressing to Hainan, Guangdong, Guangxi, and Yunnan in December. This phenomenon is strongly associated with the phenology of *Ae. albopictus* due to seasonal changes in landscape conditions [40].

The dynamics of dengue source-sink system are influenced by ecosystem landscapes, which are driven by climate conditions, vegetations, socioeconomic conditions and the repeated introductions of DENV. Since eradicating local dengue transmission (colonization, latency, spreading, and outbreaks of DENV) is the focus of public health control and prevention in China, more attention has been paid to the environmental drivers of the local dengue cases. Temperatures are a vital driver that affects the physiological processes of the mosquitoes and the DENV-vector-host nexus[41]. *Ae. aegypti* can survive in temperatures from 10 to 40 ℃ and only transmit the virus between 17.8 ℃ and 34.5 ℃ [41–43]. *Ae. albopictus* could persist in colder temperatures and spread the virus between 16.2 and 31.4 ℃ [40, 41]. The regions in the southeast Hu Line of China are affected by the East Asian monsoon, which is characterized by more rainy days and higher temperatures simultaneously in summer [44]. Our results indicate that these monsoon characteristics have a significant impact on dengue dynamics. A higher mean temperature of the driest quarter could prolong the life expectancy of mosquitoes, resulting in longer exposure durations of dengue hazards [45, 46]. The precipitation will increase the establishment of vector breeding habitats for dengue transmission [6]. Minute changes in precipitation seasonality might help maintain breeding habitats, providing sustaining dwelling niches. NDVI correlates with land cover characteristics on vegetation. Vegetation could regulate microclimatic conditions and provide suitable habitats and nectar sources for vectors [47]. According to our findings, effects driven by NDVI in early spring and mid-summer presented contrasting associations with dengue risks. Lower NVDI in mid-summer is closely linked with urbanization; higher NDVI in early spring suggests the ideal climate for mosquito biology, usually found in in South China [48]. Interestingly, we found the driving pathway and their coefficients from composite social and economic factors for population and GDP to imported dengue cases and then to the local cases, which proved the nexus among ‘social economic development-imported cases-local cases’.

The dengue dynamic system is a complex interplay between *Aedes* mosquito and the dengue virus (DENV), which is influenced by global connectivity and environmental change. Human activities can create conditions that enable the transmission of the virus (‘sparks’) and the proliferation of the mosquito population (‘culture medium’), leading to the spread of the disease (‘prairie fire’). In order to prevent and eliminate the invasion of dengue, it is important to focus not only on transit quarantine, but also on vector control. Currently, *Ae. albopictus* has spread to Shenyang, a city in northeast China at 41°N [49]. To control the abundance of *Aedes* mosquitoes in the affected areas and prevent further spread and colonization in uninfected areas, surveillance based on integrated vector control and sustainable vector control should be strengthened. This is particularly important in the context of global change and its potential impact on the spread of dengue [6, 7].

The study provides a framework that integrates landscape and invasion ecology theories, which can be applied in epidemiology to identify patterns and driving forces of the dengue source-sink system. This framework is suitable for use in other countries or regions where dengue transmission is triggered by imported cases. *Aedes* mosquitoes are the key components of the dengue source-sink system. According to a recent study, *Ae. Albopictus* is present in 177 countries, and *Ae. aegypti* is widely distributed in 155 countries [50]. The suitable distribution of these vectors is expanding due to the impact of climate change [50]. With increasing global connectivity, these countries face the risk of DENV introductions. Our framework can be applied in non-endemic countries to identify the association between oversea-imported cases and local cases in the dengue source-sink system. Furthermore, since dengue invasions exhibit a scale deduction effect, not all regions within dengue-endemic countries are endemic areas. In these regions, the relationship between domestic imported cases and local cases can be identified using our framework. This framework is helpful for understanding the international source-sink relationships and strengthening global collaborations for source control of dengue.

Our study has some limitations. Since some unreported cases of asymptomatic dengue infection, information bias could exist in the surveillance data. Due to incomplete information provided by patients, privacy issues, and potential misreporting, local cases may have been combined with domestic imported cases. Some results were focused on the average level in fifteen years, which may neglect the intrinsic fluctuations at the time-serial scale. Due to the scale effect, the results were shown the average status at certain administrative levels focused on general trends, which cannot be interpreted on local or more fine scales to avoid ecological fallacy. As the compatibility on the space–time scale with other datasets, surveillance data on vector abundance were not included to reflect the contribution of *Aedes* mosquitoes.

## Conclusions

In this study, we detected the source-sink relationships of dengue transmissions at spatial-temporal scales, confirmed the major drivers to local dengue dynamics, and updated the linkages on environmental drivers-imported cases-local cases. This study could provide a complete picture of the patterns and driving forces of dengue invasions in China, which can help to understand the priorities to prevent and control the potential threats of introduction, colonization, spread, and outbreak events under a comprehensive vision combined with invasion ecology, landscape ecology, and epidemiology.

## Supplementary Information

**Additional file** 1: **Figure S1** The distributions of China at provincial-level administrative divisions. **Table S1.** The list of the environmental drivers. **Table S2.** Augmented Dickey-Fuller test (unit root test) for local cases. **Table S3.** Critical values for test statistics of local cases. **Table S4.** Augmented Dickey-Fuller test (unit root test) for imported cases. **Table S5.** Critical values for test statistics of imported cases. **Table S6.** Lag selections for VAR. **Table S7.** Granger causality test. **Table S8.** VAR estimation results for local cases. **Table S9.** VAR estimation results for imported cases. **Table S10.** Roots of the characteristic polynomial for VAR. **Table S11.** Box-Ljung test for residuals. **Table S12.** ARCH test for Heteroscedasticity. **Table S13.** Variance decomposition for VAR. **Figure S2.** Grid search for random forest. **Table S14.** Estimation results in GAM. **Table S15.** PCA results for bioclimate variables. **Table S16.** Contributions of the BIO variables. **Table S17.** PCA results for NDVI variables. **Table S18.** Contributions of the NDVI variables. **Table S19.** PCA results for HFI and HMI variables. **Table S20.** Contributions of the Soc_Eco and HF_HM variables. **Table S21.** Model selections in SEM.

## Data Availability

The datasets used and analyzed during the current study are available from the corresponding author on reasonable request.

## Abbreviations

COVID-19: Coronavirus disease 2019
DENV: Dengue virus
NDVI: Normalized difference vegetation index
SEM: Structural equation modeling
GDD10: Growing degree days heat sum above 10 °C
CMI: Climate moisture index
GSP: Accumulated precipitation amount on growing season days TREELIM
GST: Mean temperature of the growing season TREELIM
NGD10: Number of growing degree days above 10 °C
NPP: Net primary productivity
HMI: Human modification index
HFI: Human footprint index
AIC: Akaike information criteria
VAR: Vector autoregressive model
RF: Random forest
GAM: General additive models
*OR*: Odds ratios
*CI*: Confidence intervals
PCA: Principal components analysis
PLADs: Provincial-level administrative divisions
AUC: Area under curve
SPC: Standardized path coefficient

## References

[1] Wilder-Smith A, Ooi E-E, Horstick O, Wills B (2019). Dengue. Lancet.

[2] WHO. Dengue and severe dengue: WHO newsroom. 2022. https://www.who.int/news-room/fact-sheets/detail/dengue-and-severe-dengue. Accessed 16 Jan 2023.

[3] Bhatt S, Gething PW, Brady OJ, Messina JP, Farlow AW, Moyes CL (2013). The global distribution and burden of dengue. Nature.

[4] Chen Y, Li N, Lourenço J, Wang L, Cazelles B, Dong L (2022). Measuring the effects of COVID-19-related disruption on dengue transmission in southeast Asia and Latin America: a statistical modelling study. Lancet Infect Dis.

[5] WHO. Dengue report. 2023. https://www3.paho.org/data/index.php/. Accessed 16 Jan 2023.

[6] Franklinos LHV, Jones KE, Redding DW, Abubakar I (2019). The effect of global change on mosquito-borne disease. Lancet Infect Dis.

[7] Baker RE, Mahmud AS, Miller IF, Rajeev M, Rasambainarivo F, Rice BL (2022). Infectious disease in an era of global change. Nat Rev Microbiol.

[8] Whitmee S, Haines A, Beyrer C, Boltz F, Capon AG, de Souza Dias BF (2015). Safeguarding human health in the Anthropocene epoch: report of The Rockefeller Foundation-Lancet Commission on planetary health. Lancet.

[9] Steffen W, Richardson K, Rockström J, Cornell SE, Fetzer I, Bennett EM (2015). Planetary boundaries: guiding human development on a changing planet. Science.

[10] Carlson CJ, Albery GF, Phelan A (2021). Preparing international cooperation on pandemic prevention for the Anthropocene. BMJ Glob Health.

[11] Messina JP, Brady OJ, Pigott DM, Golding N, Kraemer MUG, Scott TW (2015). The many projected futures of dengue. Nat Rev Microbiol.

[12] Findlater A, Moineddin R, Kain D, Yang J, Wang X, Lai S (2019). The use of air travel data for predicting dengue importation to China: a modelling study. Travel Med Infect Dis.

[13] Li N, Feng Y, Vrancken B, Chen Y, Dong L, Yang Q (2021). Assessing the impact of COVID-19 border restrictions on dengue transmission in Yunnan Province, China: an observational epidemiological and phylogenetic analysis. Lancet Reg Health West Pac.

[14] Chinese Center for Disease Control and Provention. The surveillance guideline for dengue. https://www.chinacdc.cn/jkzt/crb/zl/dgr/jszl_2235/. Accessed 19 Mar 2023.

[15] Karger DN, Conrad O, Böhner J, Kawohl T, Kreft H, Soria-Auza RW (2017). Climatologies at high resolution for the earth’s land surface areas. Sci Data.

[16] Center for International Earth Science Information Network - CIESIN - Columbia University. Gridded Population of the World, Version 4 (GPWv4): Population Count, Revision 11. Palisades, New York: NASA Socioeconomic Data and Applications Center (SEDAC); 2018. 10.7927/H4JW8BX5. Accessed 16 Jan 2023.

[17] Kummu M, Taka M, Guillaume JHA (2018). Gridded global datasets for Gross Domestic Product and Human Development Index over 1990–2015. Sci Data.

[18] Kennedy CM, Oakleaf JR, Theobald DM, Baruch-Mordo S, Kiesecker J. Global Human Modification of Terrestrial Systems. Palisades, New York: NASA Socioeconomic Data and Applications Center (SEDAC); 2020. 10.7927/edbc-3z60. Accessed 16 Jan 2023.

[19] Kennedy CM, Oakleaf JR, Theobald DM, Baruch-Mordo S, Kiesecker J (2019). Managing the middle: a shift in conservation priorities based on the global human modification gradient. Glob Change Biol.

[20] Mu H, Li X, Wen Y, Huang J, Du P, Su W (2022). A global record of annual terrestrial Human Footprint dataset from 2000 to 2018. Sci Data.

[21] Brewer CA, Pickle L (2002). Evaluation of methods for classifying epidemiological data on choropleth maps in series. Ann Assoc Am Geogr.

[22] Sunagawa S, Coelho LP, Chaffron S, Kultima JR, Labadie K, Salazar G (2015). Structure and function of the global ocean microbiome. Science.

[23] Genuer R, Poggi J-M, Tuleau-Malot C (2010). Variable selection using random forests. Pattern Recognit Lett.

[24] Zheng JX, Xia S, Lv S, Zhang Y, Bergquist R, Zhou XN (2021). Infestation risk of the intermediate snail host of *Schistosoma japonicum* in the Yangtze River Basin: improved results by spatial reassessment and a random forest approach. Infect Dis Poverty.

[25] Breiman L (2001). Random forests. Mach Learn.

[26] Kursa MB, Rudnicki WR (2010). Feature selection with the Boruta Package. J Stat Softw.

[27] Grace JB, Anderson TM, Seabloom EW, Borer ET, Adler PB, Harpole WS (2016). Integrative modelling reveals mechanisms linking productivity and plant species richness. Nature.

[28] Lun X, Wang Y, Zhao C, Wu H, Zhu C, Ma D (2022). Epidemiological characteristics and temporal-spatial analysis of overseas imported dengue fever cases in outbreak provinces of China, 2005–2019. Infect Dis Poverty.

[29] Bertelsmeier C, Keller L (2018). Bridgehead effects and role of adaptive evolution in invasive populations. Trends Ecol Evol.

[30] Messina JP, Brady OJ, Golding N, Kraemer MUG, Wint GRW, Ray SE (2019). The current and future global distribution and population at risk of dengue. Nat Microbiol.

[31] Sang S (2015). Dengue is still an imported disease in China: a case study in Guangzhou. Infect Genet Evol.

[32] Liu W, Hu W, Dong Z, You X (2021). Travel-related infection in Guangzhou, China, 2009–2019. Travel Med Infect Dis.

[33] Jiang L, Liu Y, Su W, Liu W, Yang Z (2021). Decreased dengue cases attributable to the effect of COVID-19 in Guangzhou in 2020. PLoS Negl Trop Dis.

[34] Qi X, Wang Y, Li Y, Meng Y, Chen Q, Ma J (2015). The effects of socioeconomic and environmental factors on the incidence of dengue fever in the Pearl River Delta, China, 2013. PLoS Negl Trop Dis.

[35] Zhu G, Liu T, Xiao J, Zhang B, Song T, Zhang Y (2019). Effects of human mobility, temperature and mosquito control on the spatiotemporal transmission of dengue. Sci Total Environ.

[36] Couper LI, Farner JE, Caldwell JM, Childs ML, Harris MJ, Kirk DG (2021). How will mosquitoes adapt to climate warming?. Elife.

[37] Rückert C, Weger-Lucarelli J, Garcia-Luna SM, Young MC, Byas AD, Murrieta RA (2017). Impact of simultaneous exposure to arboviruses on infection and transmission by *Aedes aegypti* mosquitoes. Nat Commun.

[38] Marti R, Li Z, Catry T, Roux E, Mangeas M, Handschumacher P (2020). A mapping review on urban landscape factors of dengue retrieved from earth observation data, GIS techniques, and survey questionnaires. Remote Sens.

[39] Zhang M, Huang JF, Kang M, Liu XC, Lin HY, Zhao ZY (2022). Epidemiological characteristics and the dynamic transmission model of dengue fever in Zhanjiang City, Guangdong Province in 2018. Trop Med Infect Dis.

[40] Zheng X, Zhong D, He Y, Zhou G (2019). Seasonality modeling of the distribution of *Aedes albopictus* in China based on climatic and environmental suitability. Infect Dis Poverty.

[41] Reinhold JM, Lazzari CR, Lahondère C (2018). Effects of the environmental temperature on *Aedes aegypti* and *Aedes albopictus* mosquitoes: a review. Insects.

[42] Mordecai EA, Cohen JM, Evans MV, Gudapati P, Johnson LR, Lippi CA (2017). Detecting the impact of temperature on transmission of Zika, dengue, and chikungunya using mechanistic models. PLoS Negl Trop Dis.

[43] Mordecai EA, Caldwell JM, Grossman MK, Lippi CA, Johnson LR, Neira M (2019). Thermal biology of mosquito-borne disease. Ecol Lett.

[44] Zhang J, Huan X, Lü H, Wang C, Shen C, He K (2022). Crossing of the Hu line by Neolithic population in response to seesaw precipitation changes in China. Sci Bull.

[45] Lahondère C, Bonizzoni M (2022). Thermal biology of invasive Aedes mosquitoes in the context of climate change. Curr Opin Insect Sci.

[46] Madzokere ET, Hallgren W, Sahin O, Webster JA, Webb CE, Mackey B (2020). Integrating statistical and mechanistic approaches with biotic and environmental variables improves model predictions of the impact of climate and land-use changes on future mosquito-vector abundance, diversity and distributions in Australia. Parasit Vectors.

[47] Burkett-Cadena ND, Vittor AY (2018). Deforestation and vector-borne disease: forest conversion favors important mosquito vectors of human pathogens. Basic Appl Ecol.

[48] Xu Y, Yang Y (2022). A 5 km resolution dataset of monthly NDVI product of China (1982–2020). Chin Sci Data.

[49] Yue Y, Liu Q, Liu X, Zhao N, Yin W (2022). Dengue fever in mainland China, 2005–2020: a descriptive analysis of dengue cases and *Aedes* data. Int J Environ Res Public Health.

[50] Kraemer MUG, Reiner RC, Brady OJ, Messina JP, Gilbert M, Pigott DM (2019). Past and future spread of the arbovirus vectors *Aedes aegypti* and *Aedes albopictus*. Nat Microbiol.

