# Supplementary material for: The patterns and driving forces of dengue invasions in China
Source: Infect Dis Poverty. 2023 Apr 21;12:42. doi: 10.1186/s40249-023-01093-0 (PMC10119823; doi:10.1186/s40249-023-01093-0)
Supplement: Supplementary file 1 — Additional file 1: Figure S1 The distributions of China at provincial-level administrative divisions. Table S1. The list of the environmental drivers. Table S2. Augmented Dickey-Fuller test (unit root test) for local cases. Table S3. Critical values for test statistics of local cases. Table S4. Augmented Dickey-Fuller test (unit root test) for imported cases. Table S5. Critical values for test statistics of imported cases. Table S6. Lag selections for VAR. Table S7. Granger causality test. Table S8. VAR estimation results for local cases. Table S9. VAR estimation results for imported cases. Table S10. Roots of the characteristic polynomial for VAR. Table S11. Box-Ljung test for residuals. Table S12. ARCH test for Heteroscedasticity. Table S13. Variance decomposition for VAR. Figure S2. Grid search for random forest. Table S14. Estimation results in GAM. Table S15. PCA results for bioclimate variables. Table S16. Contributions of the BIO variables. Table S17. PCA results for NDVI variables. Table S18. Contributions of the NDVI variables. Table S19. PCA results for HFI and HMI variables. Table S20. Contributions of the Soc_Eco and HF_HM variables. Table S21. Model selections in SEM. [file 40249_2023_1093_MOESM1_ESM.docx]

**Additional file 1**

Supplementary Material to: The patterns and driving forces of dengue invasion in mainland China.

**Table of Contents**

| **Page** | **Item** |
| --- | --- |
| 2 | Figure S1 The distributions of China at provincial-level administrative divisions |
| 3 | Table S1 The list of the environmental drivers |
| 4 | Table S2 The augmented Dickey-Fuller test (unit root test) for local cases |
| 4 | Table S3 Critical values for test statistics of local cases |
| 4 | Table S4 Augmented Dickey-Fuller test (unit root test) for imported cases |
| 4 | Table S5 Critical values for test statistics of imported cases |
| 4 | Table S6 Lag selections for VAR |
| 5 | Table S7 Granger causality test |
| 5 | Table S8 VAR estimation results for local cases |
| 5 | Table S9 VAR estimation results for imported cases |
| 5 | Table S10 Roots of the characteristic polynomial for VAR |
| 5 | Table S11 Box-Ljung test for residuals |
| 6 | Table S12 ARCH test for Heteroscedasticity |
| 6 | Table S13 Variance decomposition for VAR |
| 6 | Figure S2 Grid search for random forest |
| 7 | Table S14 Estimation results in GAM |
| 8 | Table S15 PCA results for bioclimate variables |
| 8 | Table S16 Contributions of the BIO variables |
| 8 | Table S17 PCA results for NDVI variables |
| 8 | Table S18 Contributions of the NDVI variables |
| 8 | Table S19 PCA results for HFI and HMI variables |
| 9 | Table S20 Contributions of the Soc_Eco and HF_HM variables |
| 9 | Table S21 Model selections in SEM |

**
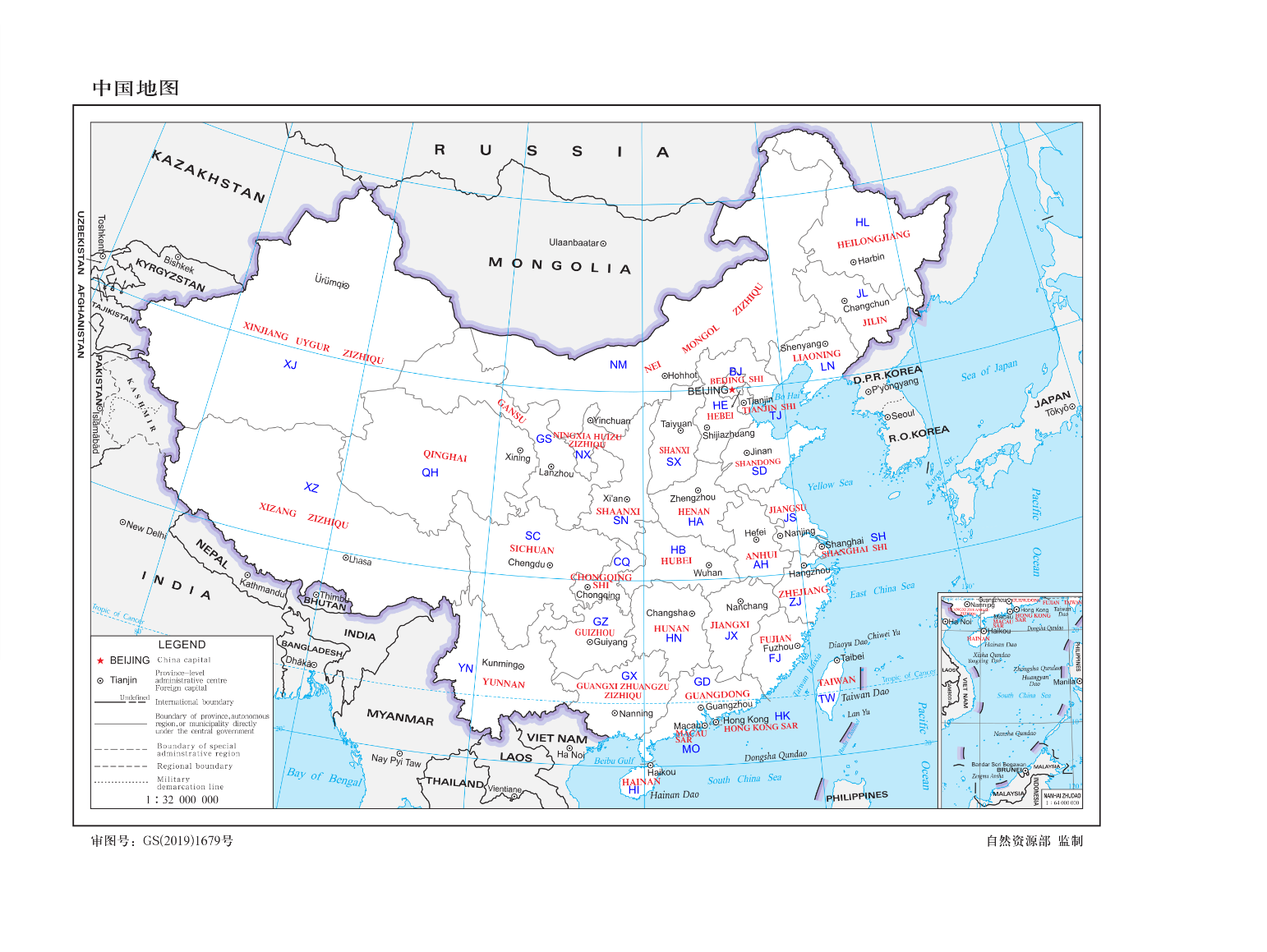
**

Figure S1 The distributions of China at provincial-level administrative divisions

(Source: http://bzdt.ch.mnr.gov.cn/browse.html?picId=%224o28b0625501ad13015501ad2bfc0281%22）

**Table S1 The list of the environmental drivers**

| **Short name** | **Long name** |
| --- | --- |
| BIO1 | Mean annual air temperature |
| BIO2 | Mean diurnal air temperature range |
| BIO3 | Isothermality |
| BIO4 | Temperature seasonality |
| BIO5 | Mean daily maximum air temperature of the warmest month |
| BIO6 | Mean daily minimum air temperature of the coldest month |
| BIO7 | Annual range of air temperature |
| BIO8 | Mean daily mean air temperatures of the wettest quarter |
| BIO9 | Mean daily mean air temperatures of the driest quarter |
| BIO10 | Mean daily mean air temperatures of the warmest quarter |
| BIO11 | Mean daily mean air temperatures of the coldest quarter |
| BIO12 | Annual precipitation amount |
| BIO13 | Precipitation amount of the wettest month |
| BIO14 | Precipitation amount of the driest month |
| BIO15 | Precipitation seasonality |
| BIO16 | Mean monthly precipitation amount of the wettest quarter |
| BIO17 | Mean monthly precipitation amount of the driest quarter |
| BIO18 | Mean monthly precipitation amount of the warmest quarter |
| BIO19 | Mean monthly precipitation amount of the coldest quarter |
| GDD10 | Growing degree days heat sum above 10°C |
| GSP | Accumulated precipitation amount on growing season days TREELIM |
| GST | Mean temperature of the growing season TREELIM |
| NGD10 | Number of growing degree days |
| NPP | Net primary productivity |
| CMI | Climate moisture index |
| NDVI | Normalized difference vegetation index in average |
| HMI | Human modification index |
| HFI | Human footprint index |
| NDVI1-12 | Normalized difference vegetation index in January to December |
| GDP | Gross domestic product |
| Population | Population counts |

**Table S2** The augmented Dickey-Fuller Test (unit root test) for local cases

|  | **Estimate** | **Std.Error** | ***t* value** | ***P*** |
| --- | --- | --- | --- | --- |
| **(Intercept)** | 0.297992 | 0.19183 | 1.553 | 0.122 |
| **z.lag.1** | -0.318489 | 0.03746 | -8.502 | < 0.001 |
| **tt** | 0.004317 | 0.00191 | 2.262 | 0.025 |
| **z.diff.lag** | 0.624178 | 0.05993 | 10.416 | < 0.001 |

model: lm(formula = z.diff ~ z.lag.1 + 1 + tt + z.diff.lag)

Residual standard error: 1.248 on 174 degrees of freedom

Multiple R-squared: 0.4443, Adjusted R-squared: 0.4347

F-statistic: 46.37 on 3 and 174 DF, p-value: < 2.2e-16

**Table S3** Critical values for test statistics of local cases

|  | **1pct** | **5pct** | **10pct** | **test-statistic** |
| --- | --- | --- | --- | --- |
| **tau3** | -3.99 | -3.43 | -3.13 | -8.5023** |
| **phi2** | 6.22 | 4.75 | 4.07 | 24.1261** |
| **phi3** | 8.43 | 6.49 | 5.47 | 36.1887** |

** P<0.01

**Table S4** The augmented Dickey-Fuller test (unit root test) for imported cases

|  | **Estimate** | **Std.Error** | ***t* value** | ***P*** |
| --- | --- | --- | --- | --- |
| **(Intercept)** | 0.29869 | 0.11817 | 2.528 | 0.012 |
| **z.lag.1** | -0.19212 | 0.04987 | -3.852 | <0.001 |
| **tt** | 0.00313 | 0.00142 | 2.205 | 0.029 |
| **z.diff.lag** | 0.05211 | 0.07747 | 0.673 | 0.502 |

lm(formula = z.diff ~ z.lag.1 + 1 + tt + z.diff.lag)

Residual standard error: 0.6776 on 174 degrees of freedom

Multiple R-squared: 0.08389, Adjusted R-squared: 0.0681

F-statistic: 5.311 on 3 and 174 DF, p-value: 0.001585

**Table S5** Critical values for test statistics of imported cases

|  | **1pct** | **5pct** | **10pct** | **test-statistic** |
| --- | --- | --- | --- | --- |
| **tau3** | -3.99 | -3.43 | -3.13 | -3.8521** |
| **phi2** | 6.22 | 4.75 | 4.07 | 5.1608** |
| **phi3** | 8.43 | 6.49 | 5.47 | 7.7374** |

*P<0.05

**Table S6** Lag selections for VAR

| **AIC(n)** | **HQ(n)** | **SC(n)** | **FPE(n)** |
| --- | --- | --- | --- |
| 2 | 2 | 2 | 2 |

**Table S7** Granger causality test

|  | **Res.df** | ***df*** | ***F* value** | ***P*** |
| --- | --- | --- | --- | --- |
| **Model_1** | 173 | — | — | — |
| **Model_2** | 175 | -2 | 6.6269 | 0.00169 |

Model 1: df_var$local ~ Lags(df_var$local, 1:2) + Lags(df_var$imported, 1:2)

Model 2: df_var$local ~ Lags(df_var$local, 1:2)

**Table S8** VAR estimation results for local cases

|  | **Estimate** | **Std. Error** | ***t*** **value** | ***P*** |
| --- | --- | --- | --- | --- |
| **local.l1** | 1.21715 | 0.0662 | 18.387 | < 0.001 |
| **imported.l1** | 0.41145 | 0.15153 | 2.715 | 0.007 |
| **local.l2** | -0.59559 | 0.06645 | -8.963 | < 0.001 |
| **imported.l2** | -0.16145 | 0.15196 | -1.062 | 0.290 |
| **const** | 0.06562 | 0.218 | 0.301 | 0.764 |

Estimation results for equation local: local = local.l1 + imported.l1 + local.l2 + imported.l2 + const

Residual standard error: 1.224 on 173 degrees of freedom

Multiple R-Squared: 0.8058, Adjusted R-squared: 0.8014

F-statistic: 179.5 on 4 and 173 DF, p-value: < 2.2e-16

**Table S9** VAR estimation results for imported cases

|  | **Estimate** | **Std. Error** | ***t* value** | ***P*** |
| --- | --- | --- | --- | --- |
| **local.l1** | 0.14412 | 0.0338 | 4.264 | < 0.001 |
| **imported.l1** | 0.73149 | 0.07736 | 9.455 | < 0.001 |
| **local.l2** | -0.20606 | 0.03393 | -6.074 | < 0.001 |
| **imported.l2** | 0.22059 | 0.07759 | 2.843 | 0.005 |
| **const** | 0.28094 | 0.1113 | 2.524 | 0.013 |

Estimation results for equation imported: imported = local.l1 + imported.l1 + local.l2 + imported.l2 + const

Residual standard error: 0.6251 on 173 degrees of freedom

Multiple R-Squared: 0.8289, Adjusted R-squared: 0.825

F-statistic: 209.6 on 4 and 173 DF, p-value: < 2.2e-16

**Table S10** Roots of the characteristic polynomial for VAR

|  | **Root_1** | **Root_2** | **Root_3** | **Root_4** |
| --- | --- | --- | --- | --- |
| **Values** | 0.9177 | 0.7954 | 0.7954 | 0.2836 |

**Table S11** Box-Ljung test for residuals

|  | ***χ*^2^** | ***df*** | ***P*** |
| --- | --- | --- | --- |
| **Local_****residuals** | 1.1684 | 4 | 0.8833 |
| **Imported_residuals** | 54.02 | 45 | 0.1678 |

**Table S12** ARCH test for Heteroscedasticity

|  | ***χ*^2^** | ***df*** | ***P*** |
| --- | --- | --- | --- |
| **Values** | 54.02 | 45 | 0.1678 |

**Table S13** Variance decomposition for VAR

|  | **Variance** | | | |
| --- | --- | --- | --- | --- |
|  | **Local** | | **Imported** | |
| **Lag order** | **Local** | **Imported** | **Local** | **Imported** |
| 1 | 1 | 0 | 0.1088974 | 0.8911026 |
| 2 | 0.9854051 | 0.01459492 | 0.2187991 | 0.7812009 |
| 3 | 0.9654833 | 0.03451675 | 0.2209071 | 0.7790929 |
| 4 | 0.9388053 | 0.06119467 | 0.1933238 | 0.8066762 |
| 5 | 0.9137849 | 0.08621514 | 0.168567 | 0.831433 |
| 6 | 0.8989955 | 0.1010045 | 0.1603423 | 0.8396577 |
| 7 | 0.8940282 | 0.10597176 | 0.1617961 | 0.8382039 |
| 8 | 0.8928378 | 0.1071622 | 0.1634817 | 0.8365183 |
| 9 | 0.8917929 | 0.10820709 | 0.1619512 | 0.8380488 |
| 10 | 0.8903544 | 0.10964561 | 0.1583611 | 0.8416389 |


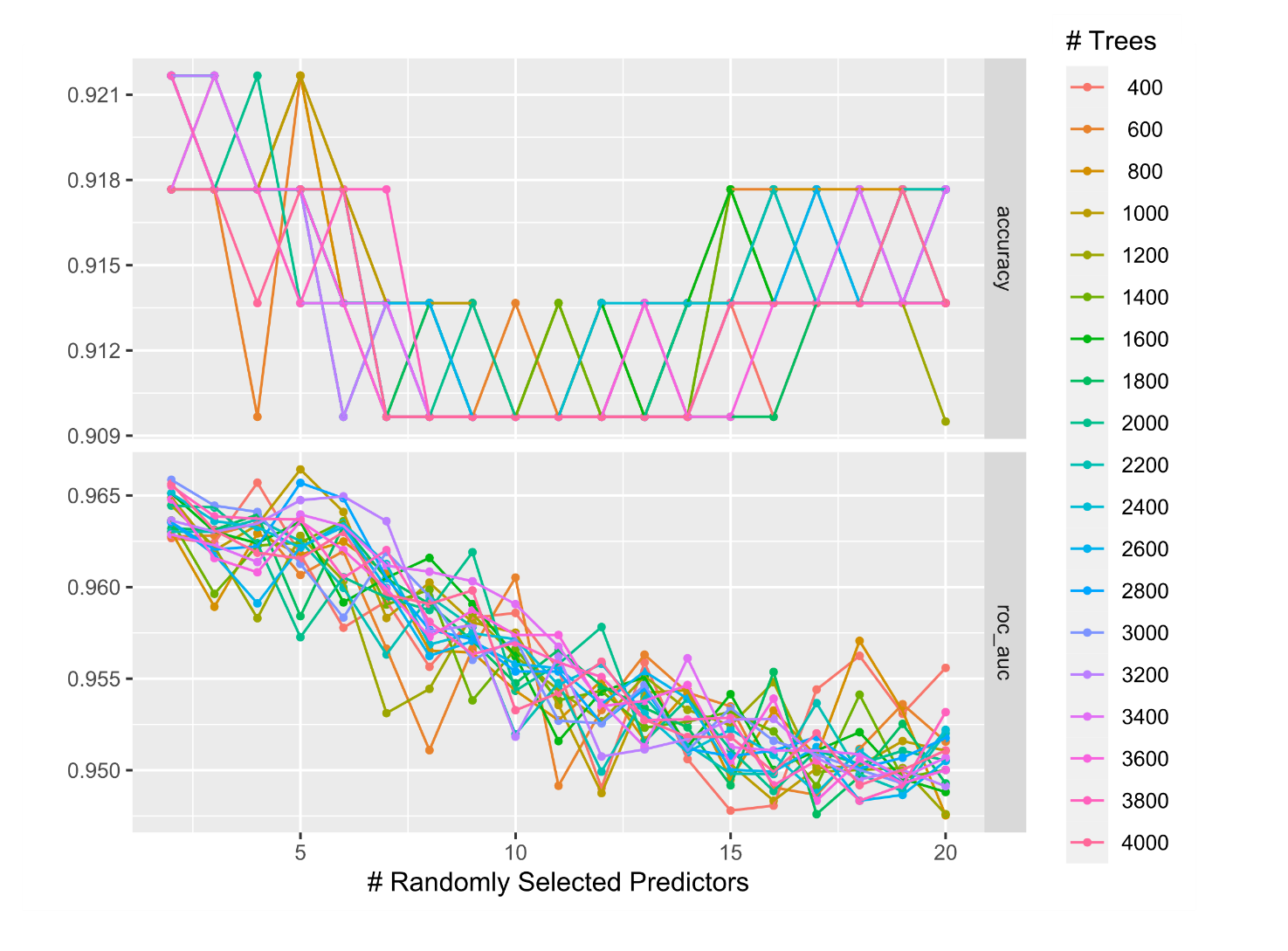


**Figure S2** Grid search for random forest

**Table S14** Estimation results in GAM

|  | **Estimate** | **Std.Error** | **t value** | ***P*** |
| --- | --- | --- | --- | --- |
| **(Intercept)** | -4.335829 | 1.191728 | -3.638 | < 0.001 |
| **s_bio8** | 0.245668 | 0.039801 | 6.172 | < 0.001 |
| **s_bio9** | 0.395302 | 0.029925 | 13.21 | < 0.001 |
| **s_bio15** | -0.070054 | 0.005799 | -12.08 | < 0.001 |
| **s_ndvi3** | 3.296747 | 1.242043 | 2.654 | 0.008 |
| **s_ndvi7** | -3.288212 | 1.384526 | -2.375 | 0.018104 |
| **s_imported** | 1.566436 | 0.050389 | 31.087 | < 0.001 |

R-sq.(adj) = 0.824

Deviance explained = 86.6%

GCV = 244.9

Scale est. = 96.787

n = 347

**Table S15** PCA results for bioclimate variables

| **Variables** | **BIO1** | **BIO2** | **BIO3** | **h2** | **u2** | **com** |
| --- | --- | --- | --- | --- | --- | --- |
| **Bio 1** | 0.39 | 0.59 | 0.70 | 0.98 | 0.02 | 2.50 |
| **Bio 10** | 0.30 | 0.13 | 0.94 | 0.98 | 0.02 | 1.20 |
| **Bio 11** | 0.40 | 0.76 | 0.48 | 0.96 | 0.04 | 2.30 |
| **Bio 12** | 0.79 | 0.55 | 0.20 | 0.97 | 0.03 | 1.90 |
| **Bio 13** | 0.64 | 0.61 | 0.19 | 0.82 | 0.18 | 2.20 |
| **Bio 14** | 0.92 | 0.17 | 0.27 | 0.95 | 0.05 | 1.30 |
| **Bio 15** | -0.76 | -0.09 | -0.22 | 0.64 | 0.36 | 1.20 |
| **Bio 16** | 0.66 | 0.64 | 0.17 | 0.88 | 0.12 | 2.10 |
| **Bio 17** | 0.92 | 0.14 | 0.26 | 0.94 | 0.07 | 1.20 |
| **Bio 18** | 0.58 | 0.67 | 0.16 | 0.81 | 0.19 | 2.10 |
| **Bio 19** | 0.92 | 0.16 | 0.24 | 0.92 | 0.08 | 1.20 |
| **Bio 2** | -0.62 | -0.39 | -0.46 | 0.75 | 0.25 | 2.60 |
| **Bio 3** | -0.25 | 0.78 | -0.34 | 0.79 | 0.22 | 1.60 |
| **Bio 4** | -0.33 | -0.91 | -0.03 | 0.94 | 0.06 | 1.30 |
| **Bio 5** | 0.21 | -0.06 | 0.94 | 0.94 | 0.06 | 1.10 |
| **Bio 6** | 0.42 | 0.72 | 0.51 | 0.95 | 0.05 | 2.50 |
| **Bio 7** | -0.39 | -0.87 | -0.16 | 0.93 | 0.07 | 1.50 |
| **Bio 8** | 0.09 | 0.15 | 0.96 | 0.95 | 0.06 | 1.10 |
| **Bio 9** | 0.49 | 0.70 | 0.46 | 0.95 | 0.05 | 2.60 |
| **CMI** | 0.84 | 0.45 | 0.06 | 0.92 | 0.08 | 1.50 |
| **GDD10** | 0.37 | 0.54 | 0.72 | 0.95 | 0.05 | 2.40 |
| **GSP** | 0.77 | 0.55 | 0.27 | 0.97 | 0.03 | 2.10 |
| **GST** | 0.14 | 0.38 | 0.82 | 0.83 | 0.17 | 1.50 |
| **NGD10** | 0.35 | 0.66 | 0.63 | 0.96 | 0.04 | 2.50 |
| **Elevation** | -0.20 | 0.16 | -0.93 | 0.92 | 0.08 | 1.20 |

**Table S16** Contributions of the BIO variables

|  | **BIO1** | **BIO2** | **BIO3** |
| --- | --- | --- | --- |
| **SS loadings** | 8.12 | 7.35 | 7.13 |
| **Proportion Var** | 0.32 | 0.29 | 0.29 |
| **Cumulative Var** | 0.32 | 0.62 | 0.90 |
| **Proportion Explained** | 0.36 | 0.33 | 0.32 |
| **Cumulative Proportion** | 0.36 | 0.68 | 1.00 |

**Table S17** PCA Results for NDVI variables

| **Variables** | **NDVI_1** | **NDVI_2** | **h2** | **u2** | **com** |
| --- | --- | --- | --- | --- | --- |
| **NPP** | 0.80 | 0.22 | 0.69 | 0.31 | 1.10 |
| **NDVI** | 0.78 | 0.62 | 1.00 | 0.00 | 1.90 |
| **NDVI 4** | 0.72 | 0.41 | 0.68 | 0.32 | 1.60 |
| **NDVI 8** | 0.12 | 0.96 | 0.94 | 0.06 | 1.00 |
| **NDVI 12** | 0.97 | 0.17 | 0.97 | 0.03 | 1.10 |
| **NDVI 2** | 0.96 | 0.18 | 0.96 | 0.04 | 1.10 |
| **NDVI 1** | 0.97 | 0.13 | 0.95 | 0.05 | 1.00 |
| **NDVI 7** | 0.13 | 0.97 | 0.96 | 0.04 | 1.00 |
| **NDVI 6** | 0.29 | 0.79 | 0.71 | 0.29 | 1.30 |
| **NDVI 3** | 0.86 | 0.32 | 0.84 | 0.16 | 1.30 |
| **NDVI 5** | 0.60 | 0.69 | 0.83 | 0.17 | 2.00 |
| **NDVI 11** | 0.92 | 0.29 | 0.94 | 0.06 | 1.20 |
| **NDVI 10** | 0.80 | 0.48 | 0.87 | 0.13 | 1.60 |
| **NDVI 9** | 0.35 | 0.89 | 0.93 | 0.07 | 1.30 |

**Table S18** Contributions of the NDVI variables

|  | **NDVI1** | **NDVI2** |
| --- | --- | --- |
| **SS loadings** | 7.40 | 4.86 |
| **Proportion Var** | 0.53 | 0.35 |
| **Cumulative Var** | 0.53 | 0.88 |
| **Proportion Explained** | 0.60 | 0.40 |
| **Cumulative Proportion** | 0.60 | 1.00 |

**Table S19** PCA results for HFI and HMI variables

| **Variables** | **Soc_Eco** | **HF_MF** | **h2** | **u2** | **com** |
| --- | --- | --- | --- | --- | --- |
| **GDP** | 0.94 | 0.27 | 0.95 | 0.05 | 1.20 |
| **Population** | 0.92 | 0.31 | 0.95 | 0.05 | 1.20 |
| **HFI** | 0.42 | 0.89 | 0.96 | 0.04 | 1.40 |
| **HMI** | 0.20 | 0.97 | 0.97 | 0.03 | 1.10 |

**Table S20** Contributions of the Soc_Eco and HF_HM variables

|  | **Soc_Eco** | **HF_HM** |
| --- | --- | --- |
| **SS loadings** | 1.94 | 1.89 |
| **Proportion Var** | 0.48 | 0.47 |
| **Cumulative Var** | 0.48 | 0.96 |
| **Proportion Explained** | 0.51 | 0.49 |
| **Cumulative Proportion** | 0.51 | 1.00 |

**Table S21** Model selections in SEM

| **Models** | **K** | **AICc** | **Delta_AICc** | **AICcWt** | **Cum.Wt** | **LL** |
| --- | --- | --- | --- | --- | --- | --- |
| **fin3** | 17 | 2255.23 | 0.00 | 0.94 | 0.94 | -1110.07 |
| **fit2** | 16 | 2260.85 | 5.61 | 0.06 | 1.00 | -1113.96 |
| **fit1** | 15 | 2276.47 | 21.24 | 0.00 | 1.00 | -1122.84 |
| **fit0** | 14 | 2509.30 | 254.07 | 0.00 | 1.00 | -1240.32 |
